# Model-based assessment of the safety of community interventions with primaquine in sub-Saharan Africa

**DOI:** 10.1186/s13071-021-05034-4

**Published:** 2021-10-09

**Authors:** Stijn W. van Beek, Elin M. Svensson, Alfred B. Tiono, Joseph Okebe, Umberto D’Alessandro, Bronner P. Gonçalves, Teun Bousema, Chris Drakeley, Rob ter Heine

**Affiliations:** 1 Department of Pharmacy, Radboud Institute for Health Sciences, Radboud University Medical Center, Nijmegen, The Netherlands; 2 Department of Pharmacy, Uppsala University, Uppsala, Sweden; 3 National Center for Research and Training on Malaria (CNRFP), Ouagadougou, Burkina Faso; 4 Department of International Public Health, Liverpool School of Tropical Medicine, Liverpool, UK; 5 Medical Research Council Unit The Gambia at the London School of Hygiene & Tropical Medicine, Faraja , The Gambia; 6 London School of Hygiene & Tropical Medicine, London, UK; 7 Department of Medical Microbiology, Radboud University Medical Center, Nijmegen, The Netherlands

**Keywords:** Primaquine, Malaria, *Plasmodium falciparum*, Mass Drug Administration, Modeling, G6PD, Anemia

## Abstract

**Background:**

Single low-dose primaquine (SLD-PQ) is recommended in combination with artemisinin-based combination therapy to reduce *Plasmodium falciparum* transmission in areas threatened by artemisinin resistance or aiming for malaria elimination. SLD-PQ may be beneficial in mass drug administration (MDA) campaigns to prevent malaria transmission but uptake is limited by concerns of hemolysis in glucose-6-phosphate dehydrogenase (G6PD)-deficient individuals. The aim of this study was to improve the evidence on the safety of MDA with SLD-PQ in a sub-Saharan African setting.

**Methods:**

A nonlinear mixed-effects model describing the pharmacokinetics and treatment-induced hemolysis of primaquine was developed using data from an adult (*n* = 16, G6PD deficient) and pediatric study (*n* = 38, G6PD normal). The relationship between primaquine pharmacokinetics and hemolysis was modeled using an established erythrocyte lifespan model. The safety of MDA with SLD-PQ was explored through Monte Carlo simulations for SLD-PQ at 0.25 or 0.4 mg/kg using baseline data from a Tanzanian setting with detailed information on hemoglobin concentrations and G6PD status.

**Results:**

The predicted reduction in hemoglobin levels following SLD-PQ was small and returned to pre-treatment levels after 25 days. G6PD deficiency (African A- variant) was associated with a 2.5-fold (95% CI 1.2–8.2) larger reduction in hemoglobin levels. In the Tanzanian setting where 43% of the population had at least mild anemia (hemoglobin < 11–13 g/dl depending on age and sex) and 2.73% had severe anemia (hemoglobin < 7–8 g/dl depending on age and sex), an additional 3.7% and 6.0% of the population were predicted to develop at least mild anemia and 0.25% and 0.41% to develop severe anemia after 0.25 and 0.4 mg/kg SLD-PQ, respectively. Children < 5 years of age and women ≥ 15 years of age were found to have a higher chance to have low pre-treatment hemoglobin.

**Conclusions:**

This study supports the feasibility of MDA with SLD-PQ in a sub-Saharan African setting by predicting small and transient reductions in hemoglobin levels. In a setting where a substantial proportion of the population had low hemoglobin concentrations, our simulations suggest treatment with SLD-PQ would result in small increases in the prevalence of anemia which would most likely be transient.

**Graphical abstract:**

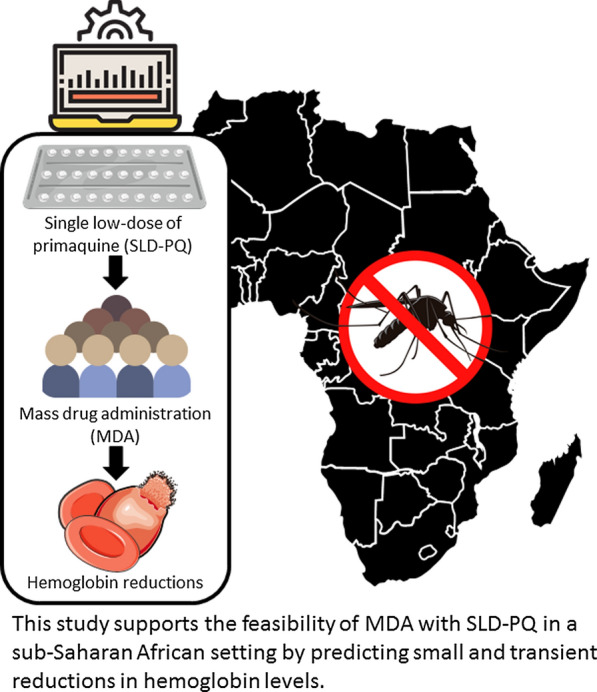

**Supplementary Information:**

The online version contains supplementary material available at 10.1186/s13071-021-05034-4.

## Background

The annual number of malaria cases is estimated at 228 million, most of them in sub-Saharan Africa [1]. *Plasmodium falciparum* is the main malaria species in this region. The transmission of malaria depends on the presence of sexual stage parasites, or gametocytes. Primaquine is the only currently available drug targeting mature *Plasmodium falciparum* gametocytes. To decrease transmission and limit development of artemisinin resistance, a single low dose of primaquine (SLD-PQ) is recommended by the World Health Organization (WHO) in combination with an artemisinin-based combination therapy [1–3]. As artemisinin resistance is emerging in sub-Saharan Africa, strategies that could limit resistance are much needed [4]. However, concerns using SLD-PQ exist because of the risk of hemolysis, especially in individuals with (severe forms of) glucose-6-phosphate dehydrogenase (G6PD) deficiency [2, 5, 6]. Primaquine-induced hemolysis may predominantly be driven by cytochrome P450 D6 (CYP2D6)-mediated metabolites [7–9].

The WHO recommends mass drug administration (MDA) for interruption of transmission in areas approaching elimination with good access to treatment and surveillance [1]. MDA consists of treating a defined population in a certain area at approximately the same time with therapeutic doses of an antimalarial. SLD-PQ at 0.25 mg per kg body weight is recommended for MDA targeting *Plasmodium falciparum* malaria [10]; this dose is considered safe even for G6PD-deficient individuals, and the WHO recommends it may be administered without testing for G6PD deficiency [2, 11–13]. However, most of the studies that assessed safety have been performed in small populations with relatively high pre-treatment hemoglobin levels. Moreover, pragmatic dosing strategies may result in some individuals receiving a higher dose than the recommended 0.25 mg/kg, which may achieve better gametocyte clearance but potentially increase the risk of hemolysis [14–16]. If co-administered with dihydroartemisinin-piperaquine (DP) instead of artemether-lumefantrine (AL), it may even be needed to use a target dose of 0.4 mg/kg to achieve the same level of gametocyte clearance [16]. This leaves the question whether some populations would still be at risk of clinically relevant hemolysis when SLD-PQ is used at population level. The aim of this study was, therefore, to predict the safety of SLD-PQ when used in MDA campaigns in a sub-Saharan African setting using population pharmacokinetic/pharmacodynamic modeling.

## Methods

### Data

Table 1 shows the characteristics of the participants included in the two studies who provided pharmacokinetic data [11, 12]. The first study was a randomized placebo-controlled trial in children from Balonghin, Burkina Faso [11]. The purpose of the study was to assess the effect of SLD-PQ (0.25 and 0.4 mg/kg) on the transmission of malaria. The study included *Plasmodium falciparum*-infected children aged 2–15 years without any malaria symptoms and normal G6PD activity. The children were treated with AL alone, AL and 0.25 mg/kg primaquine, or AL and 0.40 mg/kg primaquine. AL was given twice daily for 3 days, and primaquine or placebo was administered with the fifth dose of AL. A subset of 40 children was included in a pharmacokinetic sub-study. One blood sample was taken pre-dose, four in the first 12 h and two between 24 and 72 h after dosing. Hemoglobin concentrations were quantified using a HemoCue photometer (HemoCue AB, Angelholm, Sweden) on days 0 (pre-dosing), 2, 3, 7, 10 and 14. Two of the 40 children were excluded because of undeterminable primaquine concentrations. In total, 228 pharmacokinetic samples and 226 hemoglobin samples from 38 children were included in the analysis.

**Table 1:** Characteristics of the populations included in the analysis

Characteristic	Pediatric^a^	Adult^b^
Number of patients	38	16
G6PD deficient	No	Yes
Males, %	42.1	100
*P. falciparum* infected, %	100	69
Number treated with 0.25 mg/kg primaquine	18	10
Number treated with 0.4 mg/kg primaquine	20	6
Age in years, median (range)	9.5 (2–14)	24.5 (13–44)
Weight in kg, median (range)	23.3 (12.1–43.5)	55.4 (29.9–76.4)
Baseline Hb in g/dl, median (range)	11.7 (9.3–13.8)	13.1 (12–15.4)
Number of pharmacokinetic samples	228	97
Pharmacokinetic data BLQ, %	14.2	25.2
Number of Hb samples	226	199
CYP2D6 AS distribution, *n* (%)		
0	1 (2.63)	0 (0)
0.5	4 (10.5)	4 (25)
1	6 (15.8)	1 (6.25)
1.5	13 (34.2)	7 (43.75)
2	7 (18.4)	3 (18.75)
3	3 (7.89)	0 (0)
Missing	4 (10.5)	1 (6.25)

^a^Original data from Goncalves et al [11]. ^b^Original data from Bastiaens et al. [12].  AS: activity score; BLQ: below limit of quantification; CYP2D6: cytochrome P450 D6; Hb: hemoglobin

The second study was an open-label, randomized, dose-escalation trial in G6PD-deficient (African A- variant) adult males from Burkina Faso and The Gambia [12]. The purpose of the study was to assess the safety of SLD-PQ (0.25 mg/kg and 0.4 mg/kg) in G6PD-deficient African males. All individuals from Burkina Faso and some from The Gambia were *Plasmodium falciparum* malaria infected and asymptomatic. The participants were treated either with AL (Burkina Faso) or DP (The Gambia) alone or in combination with primaquine. Six pharmacokinetic samples were taken up to 72 h post dose from 16 participants. Randomized sampling times were allocated so that there were four samples on day 0 (day of dosing) and one each on days 1 and 2 per individual. Hemoglobin concentrations were assessed on day 0 (pre-dosing), twice daily on days 1, 2 and 3, and once daily on days 4, 5, 7, 10, 14 and 28 using self-calibrating HemoCue 201+ photometers (HemoCue AB, Angelholm, Sweden). From this second study, 97 pharmacokinetic samples and 199 hemoglobin samples were included in the analysis.

### Quantification of primaquine and genotyping of G6PD and CYP2D6

Primaquine plasma levels were quantified using liquid chromatography-mass spectrometry at two different laboratories as previously described with lower limits of quantification of 4 ng/ml and 1.14 ng/ml for the first and second study, respectively [12, 17, 18]. For the first study, G6PD status was determined using the BinaxNow rapid diagnostic test (Alere Inc., Waltham, MA, USA) as described in the original publication [11]. For the second study, G6PD status was determined using Beutler’s fluorescence spot test (R&D Diagnostics, Greece) [12]. For both studies, CYP2D6 genotype was determined with Quantstudio 12K Flex OpenArray with TaqMan assays (Thermo Fisher Scientific, Waltham, MA, USA) [12, 18].

### Pharmacokinetic/pharmacodynamic modeling

The analysis of the pharmacokinetic data and the relationship with hemoglobin concentrations over time was performed by means of nonlinear mixed-effects modeling. Model structure and estimates of previous work on the pediatric dataset were used as a starting point for the pharmacokinetic analysis [18]. Three transit compartments described the gradual absorption of primaquine. The model incorporated a well-stirred liver model [19]. Liver volume was calculated from total body weight and height [20]. A liver plasma flow of 49.5 l/h was assumed, derived from an adult total blood flow of 90 l/h and a plasma fraction of 55% in whole blood (hematocrit level 45%). Allometric scaling to a total body weight of 70 kg for volume, clearance and liver plasma flow parameters was included to account for differences in weight, with exponents of respectively 1 and 0.75 for volume and clearance parameters [21]. The bioavailability of primaquine was assumed to be 100%, and all estimated parameters are apparent oral pharmacokinetic parameters. Primaquine pharmacokinetic data below the limit of quantification were handled using the M3 method as described by Beal et al. [22].

CYP2D6 activity score (AS), a quantitative measure of phenotype, was inferred from the CYP2D6 genotype [23, 24]. The AS value for an individual can be, going from no to high activity, 0, 0.5, 1, 1.5, 2 or 3. The CYP2D6 AS was included as a covariate on the CYP2D6-mediated clearance. Quantification of the CYP2D6-mediated metabolites is difficult, and no usable pharmacokinetic data were available [21]. The same relationship between AS and CYP2D6-mediated clearance as in the model built on the pediatric data was included in the current model as alternative relationships could not be explored because of the lack of data on these metabolites [18]. The individual CYP2D6-mediated clearance was calculated as follows:

$${\text{CL}}_{\text{CYP2D6,individual}}= {\text{CL}}_{\text{CYP2D6,population}} \cdot \text{AS} \cdot {\left(\frac{\text{WT}}{70}\right)}^{0.75}$$

where the CL_CYP2D6,population_ is the estimated population CYP2D6-mediated clearance, AS is the CYP2D6 activity score, and WT is the total body weight. Missing CYP2D6 AS (11% in the pediatric study and 7% in the adult study) was imputed with the most prevalent score of 1.5. As no pharmacokinetic data on the CYP2D6-mediated metabolites were used, it was decided a priori that a single compartment with volume and clearance parameters fixed to 1 would describe the pharmacokinetics of the CYP2D6-mediated metabolites. The metabolite compartment describes a virtual CYP2D6-dependent metabolite concentration, expressed as arbitrary unit per milliliter. Carboxyprimaquine and other metabolites of primaquine were not described by the model.

**Fig. 1:**
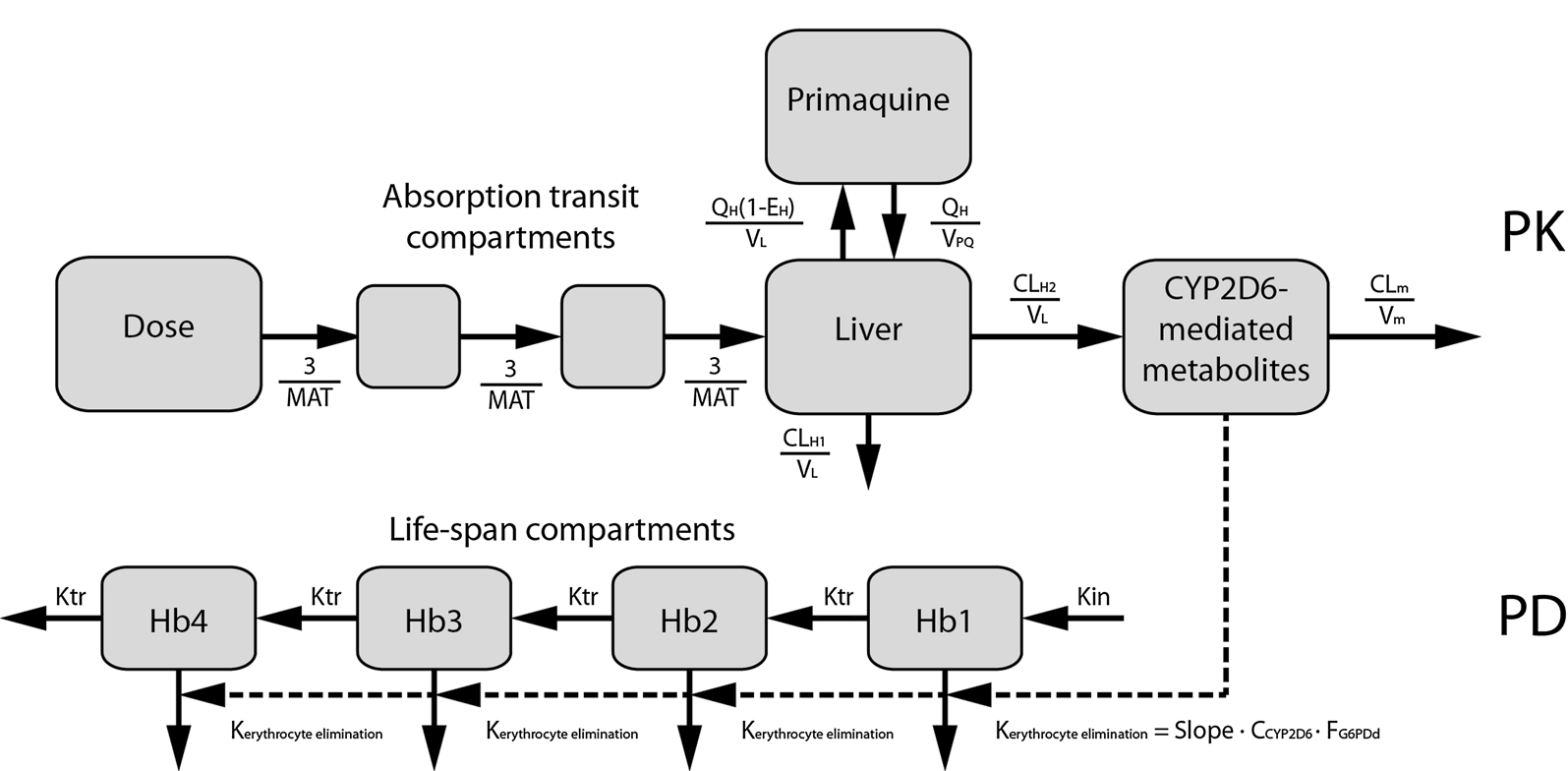
Schematic of the final pharmacokinetic/pharmacodynamic model. The pharmacokinetic model is on the upper half of the figure and the pharmacodynamic model on the lower half. *C*_CYP2D6_: concentration in the CYP2D6-mediated metabolite compartment; CL_H1_: hepatic clearance out of the system; CL_H2_: CYP2D6-mediated hepatic clearance; CL_m_: clearance of the metabolite; CYP2D6: cytochrome P450 D6; *E*_H_: hepatic extraction ratio; *F*_G6PDd_: factor by which the primaquine-induced elimination of erythrocytes is increased in G6PD-deficient individuals; Hb: hemoglobin; Kin: erythrocyte production; *K*_erythrocyte elimination_: primaquine-induced elimination of erythrocytes; Ktr: first-order rate constant defined as 4/LS where LS is the erythrocyte lifespan in hours; MAT: mean absorption time; PD: pharmacodynamic; PK: pharmacokinetic; Slope: concentration-slope effect of primaquine-induced elimination of erythrocytes; *V*_L_: liver volume; *V*_m_: volume of the metabolite compartment; *V*_PQ_: volume of the primaquine compartment

The pharmacodynamic model was developed following the pharmacokinetic model, using individual pharmacokinetic parameter estimates as input. The relationship between primaquine metabolite concentrations and hemolysis was modeled using an established erythrocyte lifespan model [25]. The erythrocyte lifespan model, shown in the lower half of Fig. 1, included four transit compartments and a concentration-slope effect describing the elimination of erythrocytes following primaquine-induced hemolysis caused by the CYP2D6-mediated metabolites. As hemoglobin is directly correlated with erythrocyte count, all lifespan compartments together make up the total hemoglobin value for an individual. The effect of G6PD deficiency on the primaquine-induced hemolysis was described by estimating a scaling factor on the concentration-slope effect. The primaquine-induced hemolysis (elimination of erythrocytes − *K*_erythrocyte elimination_) was calculated as follows:

$${K}_{\text{erythrocyte elimination}}= {C}_{\text{CYP2D6 metabolites}} \cdot \text{Slope} \cdot {F}_{\text{G6PDd}}$$

where *C*_CYP2D6 metabolites_ is the concentration in the CYP2D6-mediated metabolite compartment, Slope is the concentration-slope effect of primaquine-induced elimination of erythrocytes, and *F*_G6PDd_ is the factor by which the primaquine-induced elimination of erythrocytes is increased in G6PD-deficient individuals.

Inter-individual variability in the pharmacokinetic and pharmacodynamic parameters was assumed to be log-normally distributed. Residual variability in primaquine pharmacokinetics was implemented using a proportional error model in addition to an additive residual error which was fixed to 50% of the largest of the two lower limit of quantification values, in line with the M3 method by Beal [22]. For the pharmacodynamic model, additive, proportional and combined models were tested to describe the residual variability. The relative standard errors of the pharmacokinetic and pharmacodynamic parameters were derived from a non-parametric bootstrap with 1000 samples.

### Mass drug administration simulations

The developed model was used to explore the safety of MDA with SLD-PQ in a sub-Saharan African setting through Monte Carlo simulations. For this, we selected a large cross-sectional Tanzanian dataset that included data on age, sex, weight, hemoglobin level (g/dl) and G6PD status to create a simulation dataset [26, 27]. G6PD deficiency in this population was defined by having the hemizygous or homozygous G202A/A376G genotype, characterizing the G6PD A- variant. Children < 6 months old were removed from the dataset since we assumed they would not be included in MDA as primaquine is contraindicated for children < 6 months old and pregnant women [10]. The total number of individuals was 7672 after exclusion of children < 6 months old and individuals with missing data (568 individuals were removed). Each individual was included four times in the simulation dataset to include more combinations of CYP2D6 status and to assess the effect of inter-individual variability better. CYP2D6 AS was randomly assigned to the sampled individuals according to distributions described in the literature, resulting in probabilities of 0.026, 0.101, 0.256, 0.386, 0.209 and 0.022 for an AS of 0, 0.5, 1, 1.5, 2 and 3, respectively [28, 29].

Hemoglobin nadir was simulated for a single dose of 0.25 mg/kg or 0.4 mg/kg primaquine. We simulated doses assuming that the smallest available tablet size of primaquine is 2.5 mg and that it can be split in two (administered doses are rounded to 1.25 mg increments). The predicted prevalence of different severity classes of anemia at the nadir was assessed as a measure of safety. The severity classes of anemia consisted of mild, moderate and severe anemia and their definitions were adapted from the WHO [30]. Depending on age, sex and pregnancy status, the WHO definitions described individuals with hemoglobin levels < 11–13 g/dl as mildly anemic, individuals with hemoglobin levels < 7–11 g/dl as moderately anemic and individuals with hemoglobin levels < 7–8 g/dl as severely anemic. The complete definitions by age, sex and pregnancy status can be found in the supplementary materials (Additional file 1: Table S1).

We also explored whether the incidence of severe anemia could be limited by excluding individuals with pre-treatment hemoglobin levels below a certain threshold from dosing. Based on the hemoglobin distribution within the simulation dataset, we assessed scenarios where individuals with hemoglobin levels < 7, 7.5 and 8 g/dl were not treated with primaquine.

### Software, parameter estimation and model selection

R version 3.4.3 was used for data management, statistics and plotting [31]. Model development was performed using the nonlinear mixed-effects modeling program NONMEM version 7.4 with Pirana as an interface [32, 33]. PsN version 4.7 was used as an aid for advanced functionalities [33]. The Xpose4 R package version 4.6.1 was used for graphical visualization of the visual predictive checks (VPCs) [33]. The VPCs were performed using 1000 simulations and were prediction and variability corrected [34].

In NONMEM, the Laplacian method with interaction was used for estimation of the pharmacokinetic parameters [35]. For estimation of the pharmacodynamic parameters, the first-order conditional estimation method with interaction was used. Goodness-of-fit plots together with differences in objective function value were used to compare performances between different models. A change in objective function value of > 3.84 between two nested models was considered statistically significant (*p* < 0.05) for 1 degree of freedom.

## Results

### Pharmacokinetic modeling

Figure 1 shows the model schematic of both the pharmacokinetic and pharmacodynamic models, with the pharmacokinetic model shown in the upper half. The pharmacokinetic model for primaquine in children was successfully extended to adults by means of allometric scaling of the pharmacokinetics on body weight and re-estimation of the parameters. The estimated pharmacokinetic parameters and their uncertainty are shown in Table 2. Inter-individual variability was included on the central volume of primaquine, mean absorption time and clearance. Both the CYP2D6-mediated hepatic clearance and hepatic clearance out of the system share the same inter-individual variability. The VPC, goodness-of-fit plots and model code are included within the supplemental information (Additional file 2: Figure S1, Additional file 3: Figure S2 and Additional file 5: Model code).

**Table 2:** Final pharmacokinetic and pharmacodynamic model parameters

Parameter	Estimate	RSE^+^, CV%
*V*_PQ_, l (70 kg)	130	6.05
CL_H1_, l/h (70 kg)	25.1	8.78
CL_H2_, l/h (70 kg)	5.6	29.6
MAT, h	0.915	11.2
IIV CL^#^, CV%	46	30.6
IIV *V*_PQ_, CV%	44	26.5
IIV MAT, CV%	55	28.8
Proportional error, %	25.3	20.0
Additive error, ng/ml^a^	2	–
Lifespan, h	276	50
Slope, *C*_CYP2D6_^−1^·h^−1^	0.0012	49
*F*_G6PDd_	2.46	103
Proportional error Hb, V%	6.95	4

*C*_CYP2D6_: concentration in the compartment for CYP2D6-mediated metabolites; CL_H1_: hepatic clearance out of the system; CLH2: CYP2D6-mediated hepatic clearance; CV: coefficient of variation; *F*_G6PDd_: factor by which the primaquine-induced elimination of erythrocytes is increased in G6PD-deficient individuals; IIV: inter-individual variability; MAT: mean absorption time; RSE: relative standard error; Slope: concentration-slope effect of primaquine-induced elimination of erythrocytes; *V*_PQ_: volume of distribution for primaquine

^+^As derived from a non-parametric bootstrap with 1000 samples

^#^Both CL_H1_ and CL_H2_ share the same inter-individual variability

^a^Parameter was fixed during parameter estimation

### Pharmacodynamic modeling

The erythrocyte lifespan model schematic is shown in the lower half of Fig. 1. One concentration-slope effect describing the primaquine-induced elimination of erythrocytes was estimated for all four lifespan compartments, as we did not have data on erythrocyte populations with different ages. A lifespan of 276 h (95% CI 119–654 h) was estimated for both the G6PD-normal and -deficient individuals; separate lifespans for G6PD-normal and -deficient individuals could not be estimated reliably. The effect of G6PD deficiency on the primaquine-induced elimination of erythrocytes was estimated at a 2.46-fold increase (95% CI 1.16–8.17-fold). A proportional error model was most appropriate to describe the residual error in the pharmacodynamic model. The pharmacodynamic parameters and their uncertainty are shown in Table 2. The VPC, goodness-of-fit plots and model code are included within the supplemental materials (Additional file 2: Figure S1, Additional file 4: Figure S3 and Additional file 5: Model code).

**Fig. 2:**
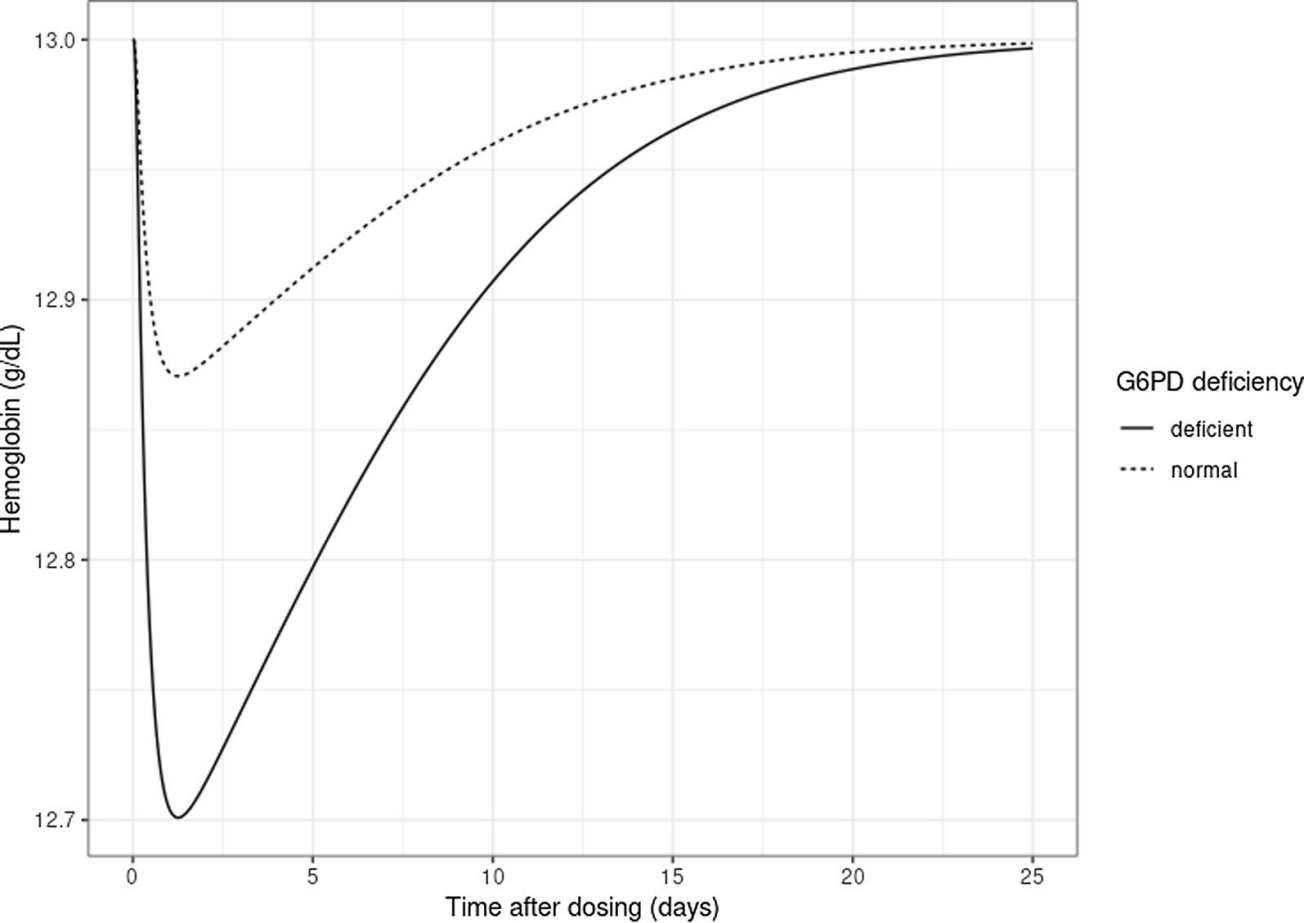
Predicted reduction in hemoglobin levels after a single dose of 0.25 mg/kg primaquine for a typical G6PD-normal and -deficient individual. A typical individual was assumed to have a weight of 70 kg, length of 170 cm, CYP2D6 activity score of 1.5 and pre-treatment hemoglobin of 13 g/dl

The predicted reduction in hemoglobin levels for a typical individual (weight 70 kg, length 170 cm, AS 1.5) with a baseline hemoglobin concentration of 13 g/dl, with and without G6PD deficiency following a single 0.25 mg/kg dose, is shown in Fig. 2. For a typical G6PD normal individual, the reduction in hemoglobin from baseline to the nadir is approximately 0.13 g/dl. For a typical G6PD-deficient individual, this reduction is 0.30 g/dl. It is predicted to take about 25 days for the hemoglobin concentration to return completely to pre-treatment values.

### Mass drug administration simulations

The median (range) of hemoglobin was 12.1 (2.0–18.8) g/dl and prevalence of G6PD deficiency was 5.51% in the simulation population. Following the linear kinetics of primaquine, the expected maximum concentration and total exposure following 0.4 mg/kg primaquine are 60% higher compared to 0.25 mg/kg primaquine. Table 3 shows the predicted median reduction in hemoglobin levels at the nadir after 0.25 mg/kg and 0.4 mg/kg primaquine for the whole population and by G6PD status. A table showing the predicted median reduction in hemoglobin levels by CYP2D6 AS group is included in the supplemental materials (Additional file 6: Table S2).

**Table 3:** Predicted median reduction in hemoglobin after 0.25 and 0.4 mg/kg primaquine

Population	Primaquine dose, mg/kg	Median reduction in Hb, g/dl	90% prediction interval of reduction in Hb, g/dl^a^
All	0.25	0.16	0.054–0.32
	0.4	0.26	0.088–0.51
G6PD normal	0.25	0.16	0.053–0.28
	0.40	0.25	0.086–0.45
G6PD deficient	0.25	0.35	0.12–0.65
	0.4	0.56	0.21–1.0

^a^Prediction interval resulting from inter-individual variability

Table 4 shows the prevalence and grade of anemia after taking 0.25 mg/kg and 0.4 mg/kg primaquine. The prevalence of anemia without any intervention was already high (43.0%) in this population and even higher (49.6%) for the G6PD-deficient individuals. After SLD-PQ of 0.25 mg/kg, an additional 3.7% of the general population was predicted to develop anemia (8.6% relative increase from pre-treatment prevalence) and an additional 0.25% to develop severe anemia specifically (9.2% relative increase from pre-treatment prevalence). Following a dose of 0.4 mg/kg primaquine, an additional 6.0% of the general population was predicted to develop anemia (14% relative increase from pre-treatment prevalence) and an additional 0.41% to develop severe anemia (15% relative increase from pre-treatment prevalence).

**Table 4:** Predicted prevalence of anemia and its severity after 0.25 and 0.4 mg/kg primaquine

Population	Dosing regimen, mg/kg	No anemia (Hb > 11–13 g/dl), %	All anemia grades (Hb < 11–13 g/dl), %	Mild anemia (Hb 7–11 to 11–13 g/dl), %	Moderate anemia (Hb 7–8 to 7–11 g/dl), %	Severe anemia (Hb < 7–8 g/dl), %
All	0	57.0	43.0	18.3	22.0	2.73
	0.25	53.3	46.7	19.6	24.1	2.98
	0.40	51.0	49.0	20.1	25.7	3.14
G6PD normal	0	57.4	42.6	18.2	21.7	2.73
	0.25	54.1	45.9	19.3	23.7	2.95
	0.40	52.0	48.0	19.8	25.1	3.09
G6PD deficient	0	50.4	49.6	19.8	27.1	2.72
	0.25	40.5	59.5	24.2	31.9	3.43
	0.40	34.8	65.2	25.2	36.1	3.96

The definitions for anemia severity are dependent on age, sex and pregnancy status, and the complete definitions can be found in Additional file 1: Table S1

**Fig. 3:**
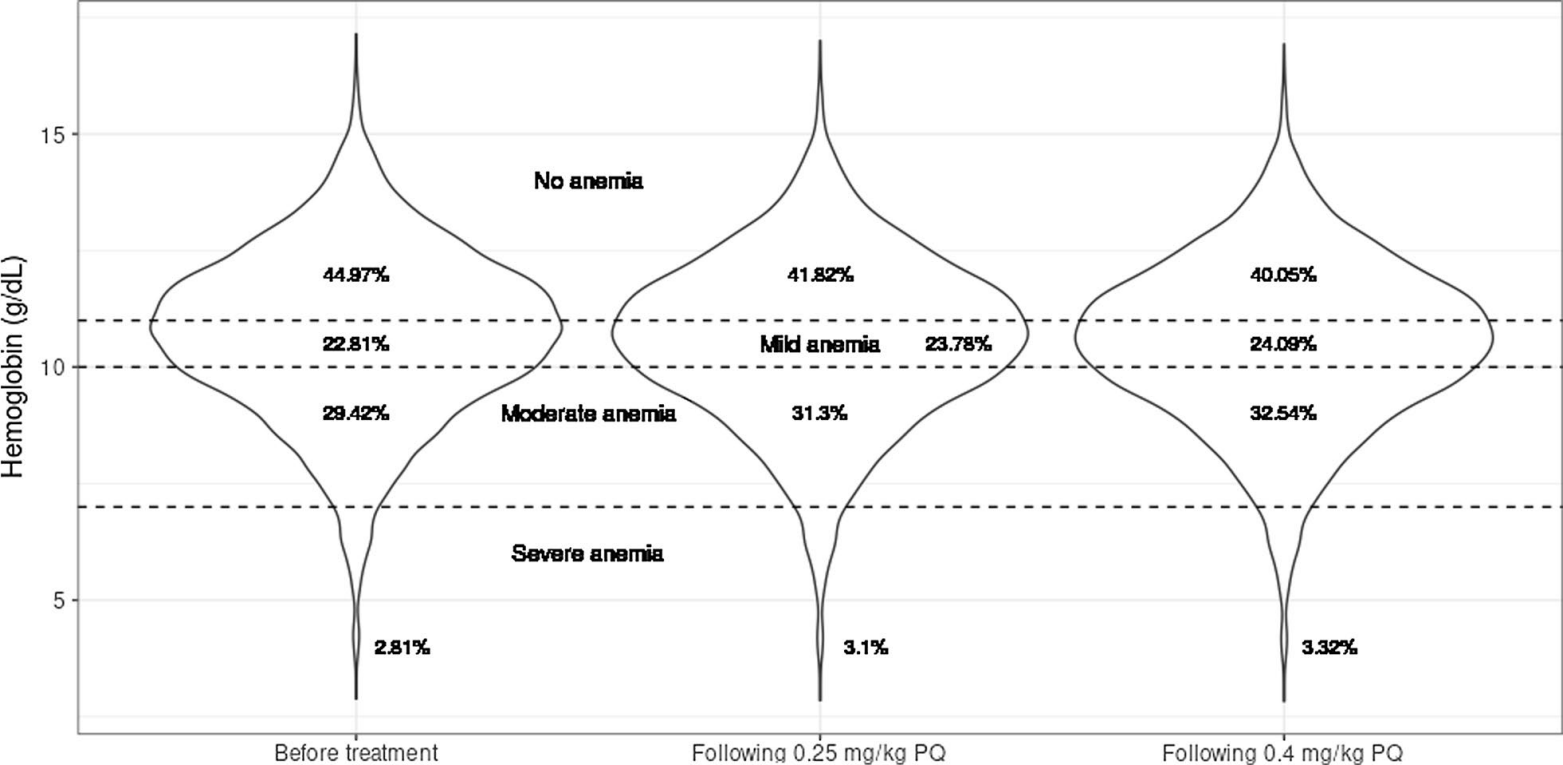
Violin plot of the simulated hemoglobin level distributions pre-treatment and following 0.25 and 0.4 mg/kg primaquine for children < 5 years of age. The dashed lines represent the cut-offs between the different groups of anemia severity. This subgroup included 26% of the total individuals in the simulation dataset of which 6.5% were G6PD deficient

The simulated hemoglobin distribution before treatment and following 0.25 and 0.4 mg/kg primaquine for children < 5 years of age is depicted in Fig. 3. The hemoglobin distributions for the other subgroups used in the anemia definition of the WHO are shown in the supplementary information (Additional file 7: Figure S4). Changes in the distribution of hemoglobin levels after SLD-PQ are minimal. Children < 5 years of age and women ≥ 15 years of age have a relatively high proportion of individuals near to and below the cut-off defining severe anemia both before and after treatment.

Excluding individuals with a pre-treatment hemoglobin value < 7, 7.5 or 8 g/dl translates into excluding 1.67%, 2.35% and 3.35% of the population, respectively (Additional file 8: Figure S5). Depending on the threshold, 41–48% of the excluded individuals are < 5 years of age compared to 26% in the general population. Table 5 shows the predicted prevalence of severe anemia after dosing with 0.25 mg/kg or 0.4 mg/kg primaquine by exclusion according to different pre-treatment hemoglobin values. For a primaquine dose of 0.25 mg/kg, the proportion of individuals transitioning from moderate to severe anemia decreased by 12%, 28% and 48% when excluding by pre-treatment hemoglobin < 7, 7.5 and 8 g/dl, respectively (i.e. from 0.25 to 0.13% of the total population for the 8 g/dl threshold). For 0.4 mg/kg primaquine, this proportion decreased by 10%, 22% and 41%, respectively. After administering 0.4 mg/kg primaquine excluding individuals with pre-treatment hemoglobin < 8 g/dl, the prevalence of severe anemia was similar to that after treating everybody with 0.25 mg/kg primaquine.

**Table 5:** Predicted prevalence of severe anemia after 0.25 or 0.4 mg/kg primaquine per dosing scenario based on pre-treatment hemoglobin level

Dosing scenario	Dosing regimen, mg/kg	Severe anemia, %
–	0	2.73
Dosing all	0.25	2.98
	0.40	3.14
Dosing Hb > 7.0 g/dl	0.25	2.95
	0.40	3.10
Dosing Hb > 7.5 g/dl	0.25	2.91
	0.40	3.03
Dosing Hb > 8.0 g/dl	0.25	2.86
	0.40	2.97

## Discussion

Concerns about primaquine safety related to hemolysis have been an obstacle to its wide implementation as the risk is at the individual level while the benefit is only gained at the population level. Contemporary safety studies provide limited information at the population level as they are typically based on selected individuals with relatively high pre-treatment hemoglobin. The present analysis describes an assessment of the relationship between primaquine concentrations and primaquine-induced hemolysis in a semi-mechanistic model with the aim of exploring the safety of an MDA campaign with SLD-PQ in a sub-Saharan African setting. The analysis shows that, post treatment, hemoglobin levels would drop in a minority of individuals to levels below the pre-defined threshold defining (severe) anemia but that this effect is transient. G6PD-deficient individuals were found to be more at risk because of the increased hemolytic effect of primaquine.

The estimated pharmacokinetic parameters were similar between our model and the model built on the pediatric data [18]. The erythrocyte lifespan was estimated at 276 h, or 11.5 days, which is short compared to what is expected in healthy individuals (70–140 days) [36]. However, considering the facts that malaria infection itself drastically reduces erythrocyte lifespan (16–84 days) and that in individuals taking oxidative medication, like primaquine, the lifespan may even be shortened to 2.5–5 days, we consider our findings in line with the literature [37, 38]. Although one may argue that our population predictions for safety in a healthy population may not necessarily be representative as they are based on estimates from mostly malaria-infected patients of which many were G6PD deficient, we consider our predictions a “worst case scenario.”

The MDA simulations showed an increase in the prevalence of anemia (hemoglobin < 11–13 g/dl) after a single dose of both 0.25 mg/kg and 0.4 mg/kg primaquine of 3.7% (8.6% relative increase) and 6.0% (14% relative increase), respectively. The increase in the prevalence of anemia was determined at nadir hemoglobin levels following primaquine treatment and was shown to be transient. After reaching the nadir following primaquine administration, the hemoglobin levels quickly recovered to return to baseline again after 25 days such that the reported increases in prevalence of anemia are present for a limited time [12, 39, 40]. The time to hemoglobin recovery was predicted to be independent of G6PD status or hemoglobin levels. The rapid recovery of hemoglobin levels also suggests that consecutive rounds of MDA with at least 1 month in between, as typically implemented [41, 42], would be unlikely to affect the prevalence of anemia.

Severe anemia is of most concern clinically, and whilst the relative increase in the prevalence of severe anemia was similar to that of any anemia grade in our Tanzanian setting, the absolute increase was much lower (0.25% vs 3.7%). This suggests that post-SLD-PQ hemoglobin levels in a small but non-negligible number of individuals might drop below our severe anemia threshold of hemoglobin < 7–8 g/dl. Again, our predictions suggested this drop would be transient. Acknowledging that individuals with low hemoglobin are at higher risk of developing severe anemia, we investigated the exclusions of individuals with hemoglobin levels below specific thresholds. Children < 5 years of age and women ≥ 15 years of age were most likely to have low hemoglobin levels in our simulations and were most at risk to develop severe anemia. As expected, we predicted that the prevalence of individuals with severe anemia is reduced by not treating individuals with low pre-treatment hemoglobin levels (< 7, < 7.5 or < 8 g/dl) although measuring hemoglobin on a large scale may be logistically challenging. Similarly, we predicted that by excluding individuals with hemoglobin values < 8 g/dl, 0.4 mg/kg primaquine can be used instead of 0.25 mg/kg without increasing the prevalence of severe anemia. Higher doses of SLD-PQ may be useful in the absence of specific low-dose or pediatric formulations of the drug.

It is important to acknowledge that we did not account for females who are heterozygous for G6PD deficiency genes as we did not have the data to do so. Heterozygous females have been described to present with a wide range of G6PD activity. In our analysis we decided to include the heterozygous females in the G6PD normal group as the majority will have a G6PD activity which is closer to normal than deficient activity [43, 44]. We performed a sensitivity analysis by simulating MDA in which heterozygous females were included in the G6PD-deficient group. In this simulation, 17.2% of the total population was defined to be G6PD deficient instead of 5.5% in the main analysis. The impact on the prevalence of anemia following SLD-PQ is generally negligible, e.g. the prevalence of severe anemia following 0.25 mg/kg primaquine increases from 2.98% to 3.02%. The full results of the sensitivity analysis can be found in the supplementary materials (Additional file 9: Table S3).

The data used to develop the model did not include children < 2 years of age and our model does not include any enzymatic maturation factors on either the pharmacokinetics or pharmacodynamics. As MDA is recommended to include children from the age of 6 months, the uncertainty in the extrapolation from our model to these younger children should be carefully considered. However, maturation of CYP2D6-dependent metabolism is not thought to play a role at this age [45], and recent findings support the safety of primaquine in young children [46]. A further limitation is that this study included only individuals who were G6PD normal or G6PD deficient with the African A- variant, the dominant variant in sub-Saharan Africa [29]. As the African A- variant is not the most severe variant of G6PD deficiency, this warrants caution for extrapolating to regions where more severe variants are prevalent. For example, individuals with the Mediterranean G6PD-deficiency variant have much lower G6PD enzyme activity compared to the African A- variant and subsequently are more at risk of severe hemolytic events. It should also be acknowledged that the Tanzanian dataset we used for MDA simulations may differ from other sub-Saharan African populations. For example, the simulation data were collected from villages at different altitudes, and whilst this encompasses a range of malaria endemicities, population hemoglobin levels will differ from other areas. As with other similar chemotherapeutic interventions, the epidemiology of malaria and co-infections would need to be considered in designing and implementing an MDA with SLD-PQ. Furthermore, our simulations were based on the likely availability of lower strength tablets produced at good manufacturing practice standards in the near future. These formulations are not currently available, and until then it will be more difficult to dose at the same precision as in our simulations. Lastly, it is important to emphasize that we have been cautious by using quite conservative definitions of anemia compared to other commonly used definitions, which further supports the safety of MDA with SLD-PQ in this population [47].

## Conclusions

We predict a small drug concentration-dependent increase in hemolysis following primaquine administration, which disappears completely after 25 days. G6PD deficiency was associated with a 2.5-fold larger reduction in hemoglobin levels. MDA with SLD-PQ is predicted to result in a small and transient relative increase in the prevalence of anemia. Children < 5 years of age and women ≥ 15 years of age were found to have a higher chance to have low pre-treatment hemoglobin. Individuals with low pre-treatment hemoglobin are at increased risk of severe anemia but this is also expected to be transitory. By exclusion from dosing of individuals with low pre-treatment hemoglobin, the incidence of severe anemia after SLD-PQ treatment could be limited. This study supports the feasibility of MDA with SLD-PQ in a sub-Saharan African setting where anemia may be common.

## Supplementary Information

**Additional file 1: Table S1.** Definition of anemia according the World Health Organization.

**Additional file 2: Figure S1.** Visual predictive checks for the final pharmacokinetic and pharmacodynamic models.

**Additional file 3: Figure S2.** Goodness-of-fit plots for the pharmacokinetic model.

**Additional file 4: Figure S3.** Goodness-of-fit plots for the pharmacodynamic model.

**Additional file 5: Model code.** NONMEM control stream of the pharmacokinetic/pharmacodynamic model.

**Additional file 6: file 6: Table S2. **Predicted median reduction in hemoglobin after 0.25 and 0.4 mg/kg primaquine stratified by CYP2D6 activity score group.

**Additional file 7: Figure S4.** Simulated hemoglobin distributions before and after treatment.

**Additional file 8: Figure S5.** Distribution of observed pre-treatment hemoglobin levels in the simulation dataset.

**Additional file 9: Table S3.** Predicted prevalence of anemia and its severity after 0.25 and 0.4 mg/kg primaquine when heterozygous females are included in the G6PD-deficient group.

## Data Availability

The datasets used during the model development can be requested from the original authors through the Worldwide Antimalarial Resistance Network repository using their PubMed IDs (27010,542 and 26952094), https://app-live.wwarn.org/DataInventoryExplorer/#1. **Attribution of graphical abstract resources:** Computer simulation icon designed by Srip from Flaticon—www.flaticon.com/authors/srip. Population icon icon designed by Smashicons from Flaticon—www.flaticon.com/authors/smashicons. Pill blister pack, map of Africa and erythrocytes icons designed by Servier Medical Art—smart.servier.com. Mosquito icon designed by Freepik: www.freepik.com.

## Abbreviations

SLD-PQ: Single low dose of primaquine
WHO: World Health Organization
G6PD: Glucose-6-phosphate dehydrogenase
CYP2D6: Cytochrome P450 D6
MDA: Mass drug administration
DP: dihydroartemisinin-piperaquine
AL: Artemether-lumefantrine
AS: Activity score
VPCs: Visual predictive checks
