# Supplementary material for: Model-based assessment of the safety of community interventions with primaquine in sub-Saharan Africa
Source: Parasit Vectors. 2021 Oct 9;14:524. doi: 10.1186/s13071-021-05034-4 (PMC8502297; doi:10.1186/s13071-021-05034-4)
Supplement: Supplementary file 1 — Additional file 1: Table S1. Definition of anemia according the World Health Organization. [file 13071_2021_5034_MOESM1_ESM.pdf]

Additional file 1: Table S1. Definition of anemia according the World Health Organization.

Definition of anemia according to the World Health Organization (1)

		Anemia*		
Population	No anemia*	Mild	Moderate	Severe
Children 6-59 months of age	≥11.0	10.0-10.9	7.0-9.9	<7.0
Children 5-11 years of age	≥11.5	11.0-11.4	8.0-10.9	<8.0
Children 12-14 years of age	≥12.0	11.0-11.9	8.0-10.9	<8.0
Non-pregnant women[#]	≥12.0	11.0-11.9	8.0-10.9	<8.0
Pregnant women	≥11.0	10.0-10.9	7.0-9.9	<7.0
Men[#]	≥13.0	11.0-12.9	8.0-10.9	<8.0

*hemoglobin in g/dL
[#]15 years of age and above

1. World Health Organisation. Haemoglobin concentrations for the diagnosis of anaemia and assessment of severity 2011 [Available from: https://www.who.int/vmnis/indicators/haemoglobin.pdf].
