# Supplementary material for: Model-based assessment of the safety of community interventions with primaquine in sub-Saharan Africa
Source: Parasit Vectors. 2021 Oct 9;14:524. doi: 10.1186/s13071-021-05034-4 (PMC8502297; doi:10.1186/s13071-021-05034-4)
Supplement: Supplementary file 2 — Additional file 2: Figure S1. Visual predictive checks for the final pharmacokinetic and pharmacodynamic models. [file 13071_2021_5034_MOESM2_ESM.pdf]

Additional file 2: Figure S1. Visual predictive checks for the final pharmacokinetic and pharmacodynamic models.

Visual predictive checks for the final (A) pharmacokinetic and (B) pharmacodynamic models. The visual predictive checks are based on 1000 simulations and are prediction corrected. The solid lines represent the mean of the observed concentrations and the dashed lines represent the 2.5th and 97.5th percentiles. The shaded areas represent the 95% confidence interval of the 2.5th, 50th, and 97.5th percentiles of the simulated concentrations.
LLOQ, lower limit of quantification

A

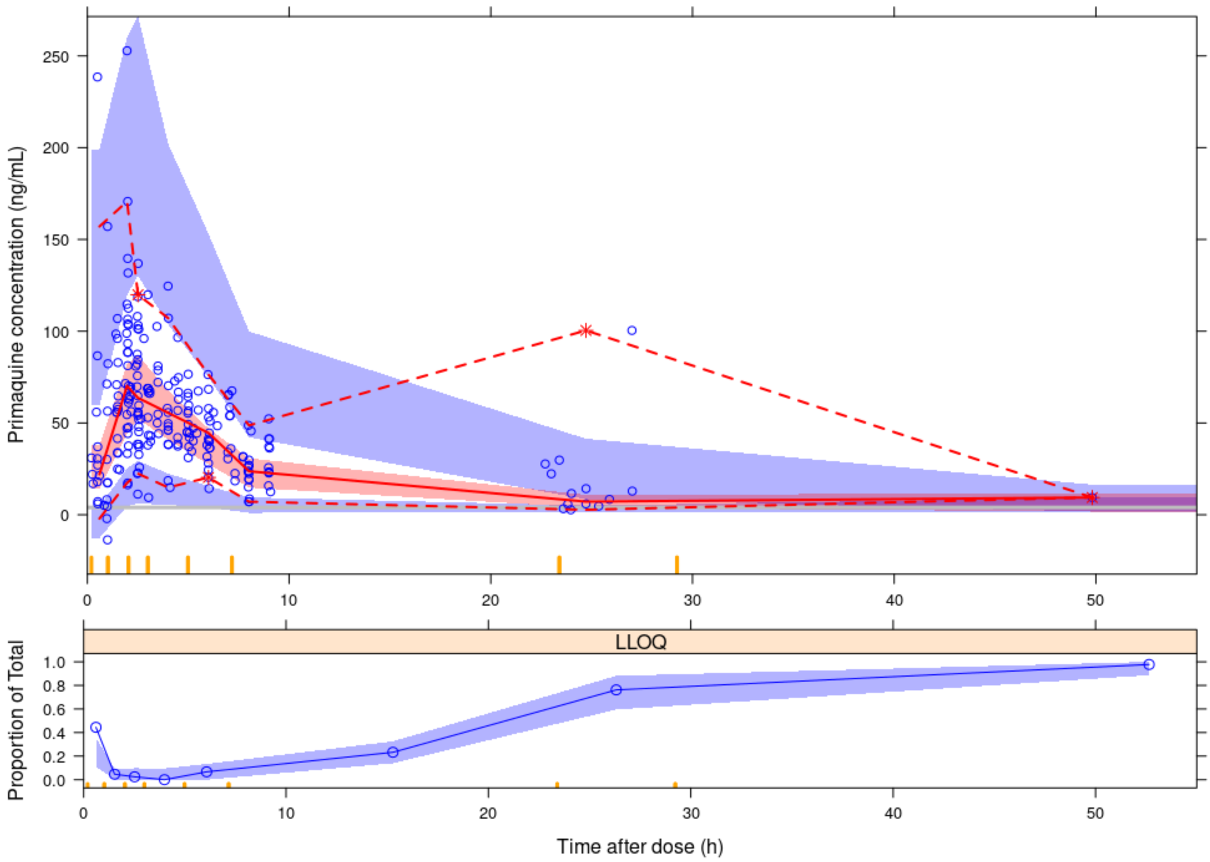

B

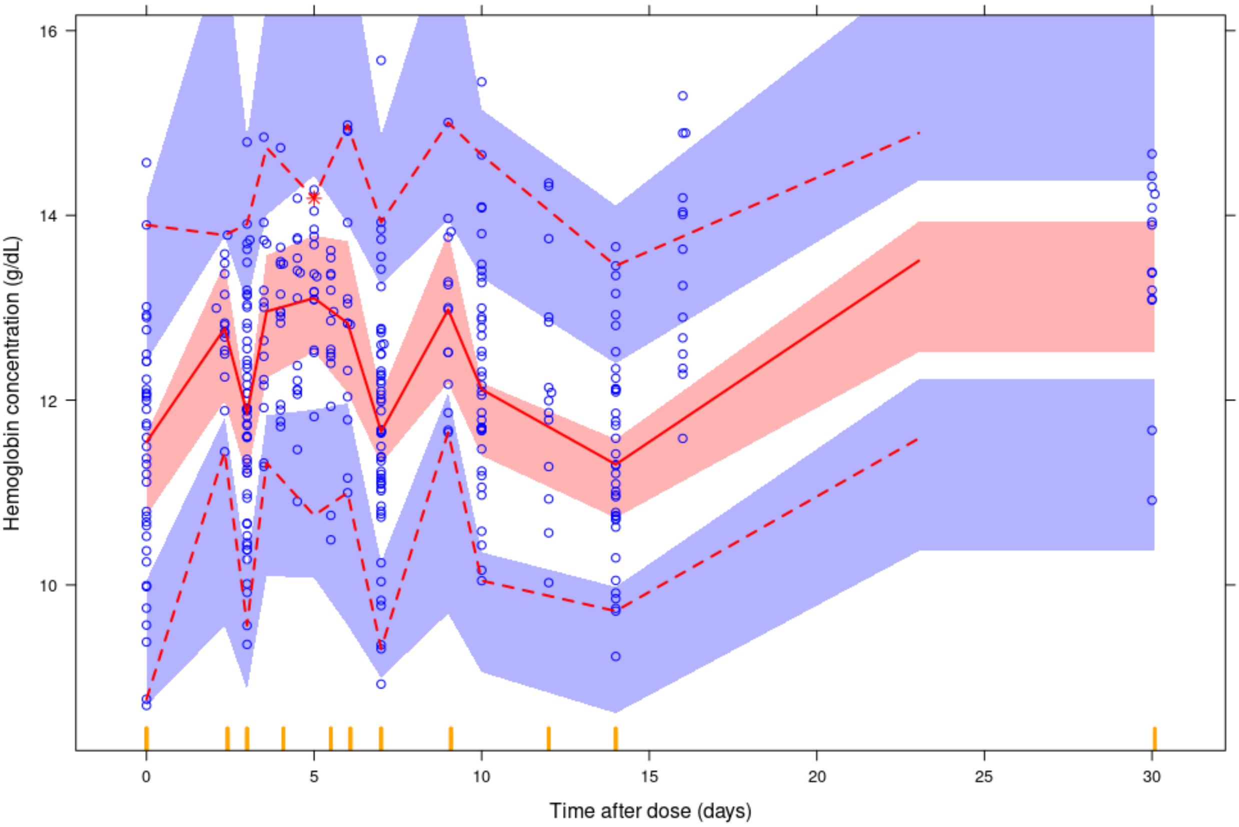
16
14
12
10
Hemoglobin concentration (g/dL)
0
5
10
15
20
25
30
Time after dose (days)
