# Supplementary figures and images for: Model-based assessment of the safety of community interventions with primaquine in sub-Saharan Africa
Source: Parasit Vectors. 2021 Oct 9;14:524. doi: 10.1186/s13071-021-05034-4 (PMC8502297; doi:10.1186/s13071-021-05034-4)

**Additional file 3: Figure S2. Goodness-of-fit plots for the pharmacokinetic model.**

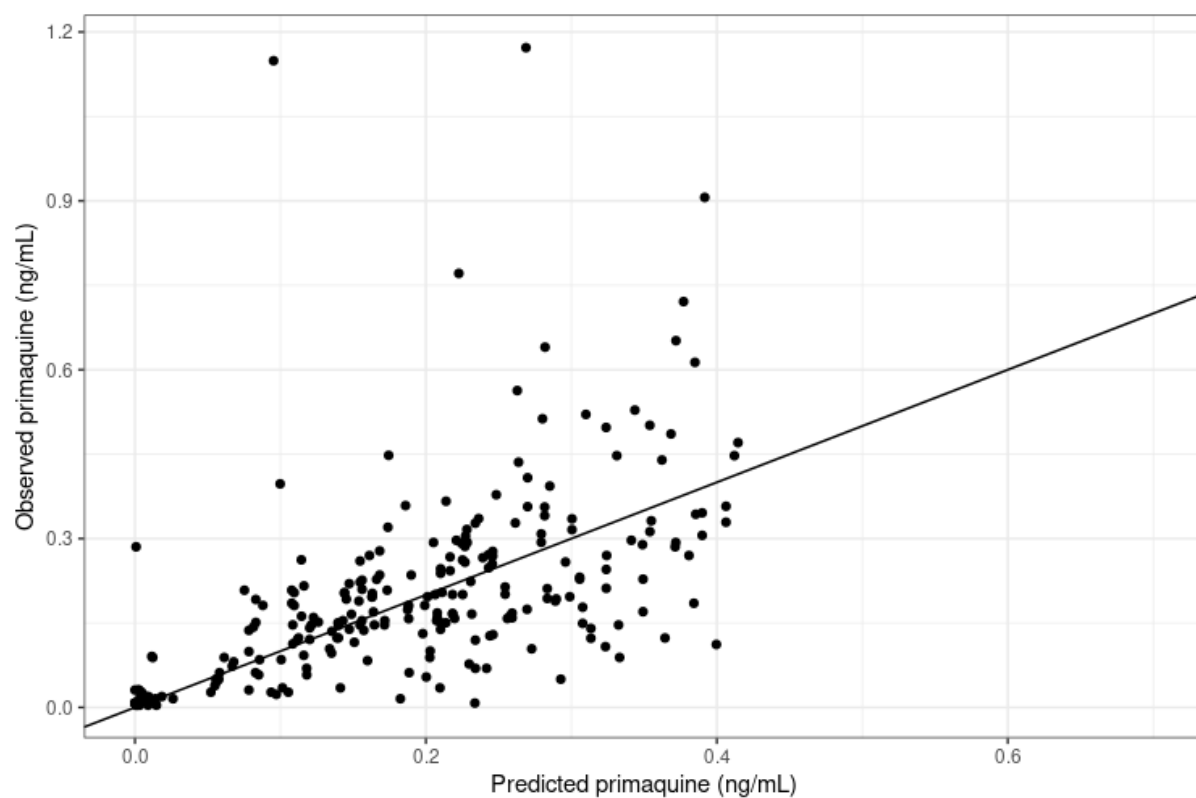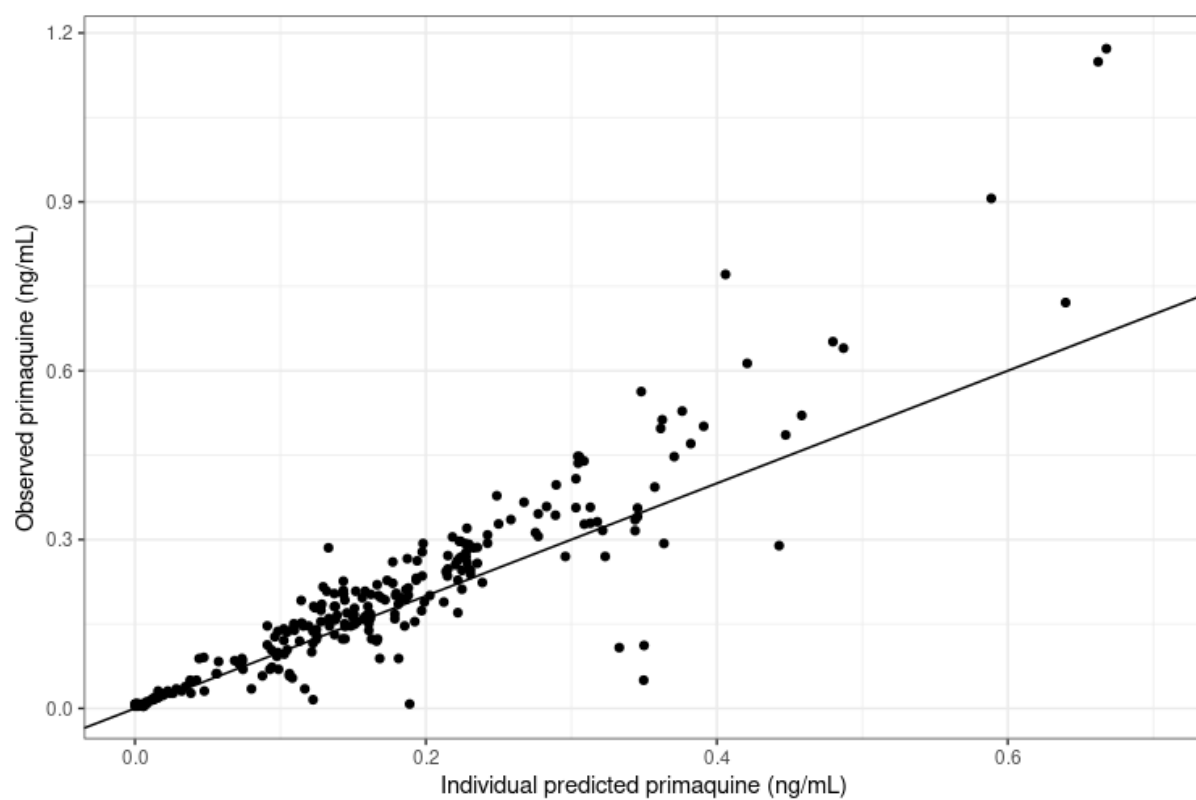

Supplement: Supplementary file 3 — Additional file 3: Figure S2. Goodness-of-fit plots for the pharmacokinetic model. [file 13071_2021_5034_MOESM3_ESM.pdf]

**Additional file 4: Figure S3. Goodness-of-fit plots for the pharmacodynamic model.**

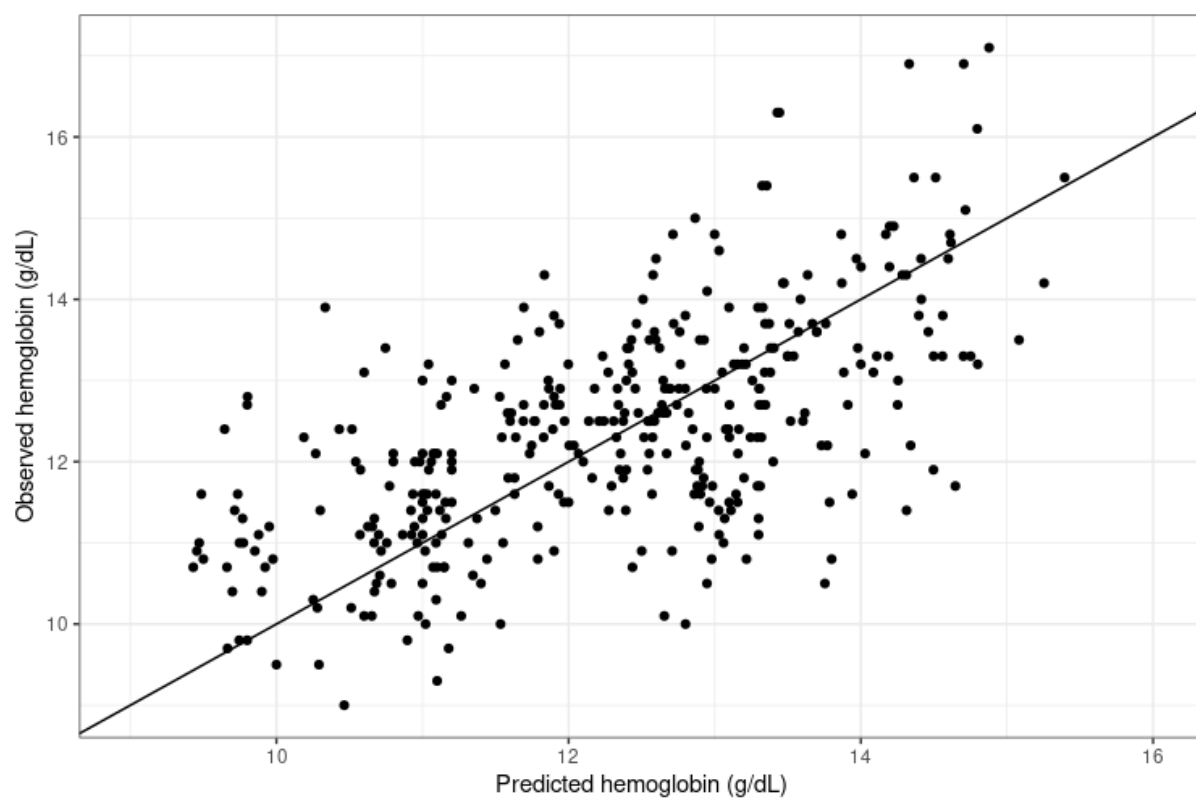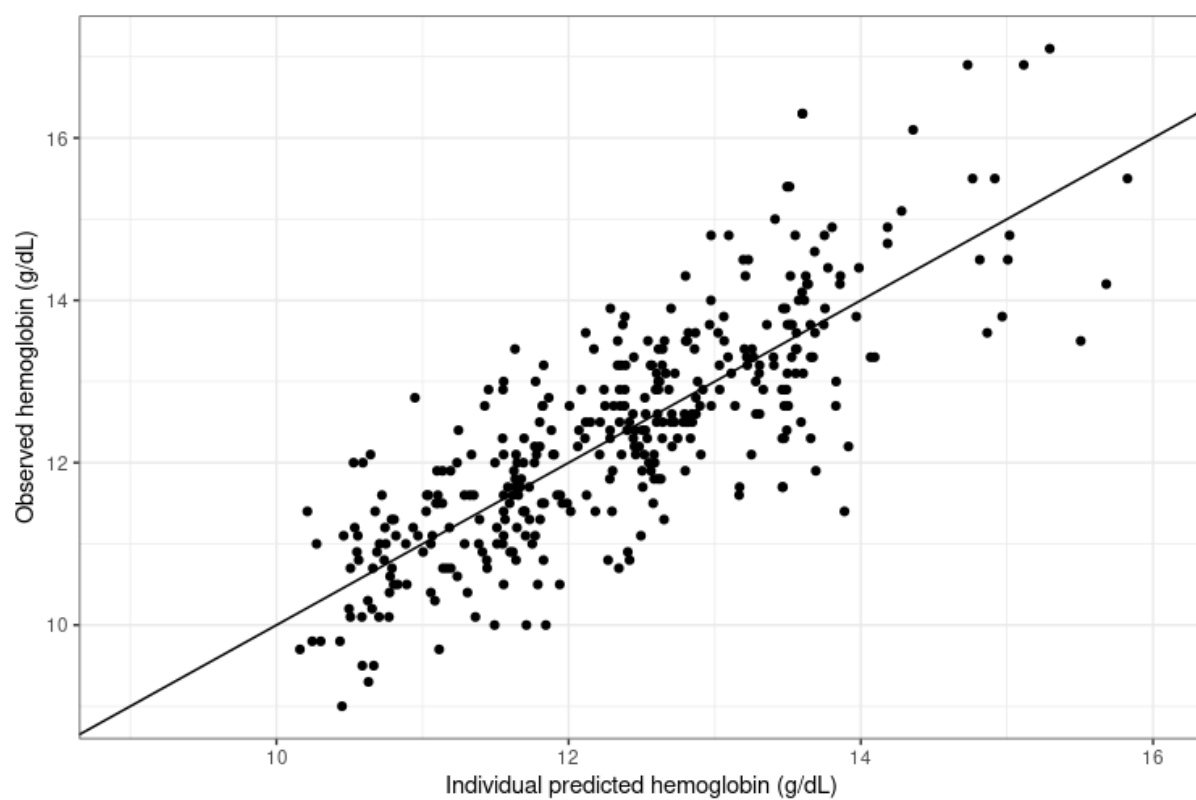

Supplement: Supplementary file 4 — Additional file 4: Figure S3. Goodness-of-fit plots for the pharmacodynamic model. [file 13071_2021_5034_MOESM4_ESM.pdf]
