# Supplementary material for: Model-based assessment of the safety of community interventions with primaquine in sub-Saharan Africa
Source: Parasit Vectors. 2021 Oct 9;14:524. doi: 10.1186/s13071-021-05034-4 (PMC8502297; doi:10.1186/s13071-021-05034-4)
Supplement: Supplementary file 5 — Additional file 5: Model code. NONMEM control stream of the pharmacokinetic/pharmacodynamic model. [file 13071_2021_5034_MOESM5_ESM.pdf]

Additional file 5: Model code. NONMEM control stream of the pharmacokinetic/pharmacodynamic model.

```
$PROB PD
$INPUT ID TIME DV EVID CMT DOSE AMT BLQ SITE AGE HT WT SEX AS PEDI HB HBBASE ICLINT1
ICLINT2 IV IMAT

; AMOUNTS IN UMOL
; CONCENTRATIONS IN UMOL/L
; TIME IN H
; VOLUMES IN L
; HB in g/dL
; ICLINT1, ICLINT2, IV and IMAT are the individual values for these parameters from the PK model

$DATA      PQ_PD_DATASET.csv IGNORE=@
$SUBROUTINE ADVAN13 TOL=6
$MODEL     COMP=(DOSE) COMP=(TRAN1) COMP=(TRAN2) COMP=(LIVER)
           COMP=(PRIMA) COMP=(HB1) COMP=(HB2) COMP=(HB3) COMP=(HB4) COMP=(METAB)

$PK
; PRIMAQUINE PK
F1      =    1                              ; BIOAVAILABILITY
MAT     =    IMAT                           ; MEAN ABSORPTION TIME
KA      =    3/MAT                          ; ABSORPTION
V       =    IV                             ; VOLUME OF DISTRIBUTION CENTRAL

CLINT1  =    ICLINT1
CLINT2  =    ICLINT2
CLINT   =    CLINT1+CLINT2

BSA     =    ((WT**0.425)*(HT**0.725))*0.007184
VL      =    0.722*(BSA**1.176)
QHP     =    90*0.55*((WT/70)**0.75)        ; HEPATIC PLASMA FLOW L/H
EH      =    CLINT/(QHP+CLINT)
EH1     =    CLINT1*EH*(1/CLINT)
EH2     =    CLINT2*EH*(1/CLINT)

CLH1    =    EH1*QHP
CLH2    =    EH2*QHP
CLH     =    EH*QHP

S5      =    V

VM      =    1*(WT/70)
CLM     =    1*((WT/70)**0.75)

; PD
BASE    =    HBBASE * EXP(ETA(1)*THETA(4))
LS      =    THETA(1)                       ;   LIFESPAN
KTR     =    4/LS
KIN     =    BASE/LS
```

```
IF(G6PD.EQ.0) SLOPE     =           THETA(2)                      ; SLOPE FOR G6PD NORMAL
IF(G6PD.EQ.1) SLOPE     =           THETA(2) * THETA(3)      ; SLOPE FOR G6PD DEFICIENCT

A_0(6)          =       (BASE/4)
A_0(7)          =       (BASE/4)
A_0(8)          =       (BASE/4)
A_0(9)          =       (BASE/4)

; MASS TRANSPORT
K12  = KA
K23  = KA
K34  = KA
K40  = CLH1/VL
K45  = (QHP*(1-(EH1+EH2)))/VL
K54  = QHP/V
K100= CLM/VM
K410= CLH2/VL

$DES
C5              =       A(5)/V
EFF             =       SLOPE * (A(10)/VM)
DADT(1)         =       -K12*A(1)                                                       ; DOSE
DADT(2)         =       -K23*A(2) +K12*A(1)                                             ; TRAN1
DADT(3)         =       -K34*A(3) +K23*A(2)                                             ; TRAN2
DADT(4)         =       -K45*A(4) -K40*A(4) -K410*A(4) +K34*A(3) +K54*A(5)              ; LIVER
DADT(5)         =       -K54*A(5) +K45*A(4)                                             ; PRIMA
DADT(6)         =       -KTR*A(6) +KIN -EFF*A(6)                                        ; HB1
DADT(7)         =       -KTR*A(7) +KTR*A(6) -EFF*A(7)                                   ; HB2
DADT(8)         =       -KTR*A(8) +KTR*A(7) -EFF*A(8)                                   ; HB3
DADT(9)         =       -KTR*A(9) +KTR*A(8) -EFF*A(9)                                   ; HB4
DADT(10)        =       -K100*A(10) +K410*A(4)                                          : METAB
HB              =       A(6)+A(7)+A(8)+A(9)                                             ; HB

$ERROR
AA6             =       A(6)
AA7             =       A(7)
AA8             =       A(8)
AA9             =       A(9)

IPRED           =       A(6)+A(7)+A(8)+A(9)
Y               =       IPRED + (IPRED * EPS(1)*THETA(4))

$THETA
 (0,276)        ; 1 LS
 (0,0.0012)     ; 2 SLOPE
 (1,2.46)       ; 3 GD6PD-SLOPE
 (0,0.0695)     ; 4 PROP ERR HB


$OMEGA
 1 FIX          ; 1 HB base
$SIGMA  1 FIX   ; 1 PROP ERR HB
```
