# Supplementary material for: Model-based assessment of the safety of community interventions with primaquine in sub-Saharan Africa
Source: Parasit Vectors. 2021 Oct 9;14:524. doi: 10.1186/s13071-021-05034-4 (PMC8502297; doi:10.1186/s13071-021-05034-4)
Supplement: Supplementary file 6 — Additional file 6: file 6: Table S2. Predicted median reduction in hemoglobin after 0.25 and 0.4 mg/kg primaquine stratified by CYP2D6 activity score group. [file 13071_2021_5034_MOESM6_ESM.pdf]

Additional file 6: Table S2. Predicted median reduction in hemoglobin after 0.25 and 0.4 mg/kg primaquine stratified by CYP2D6 activity score group.

CYP2D6 activity score	Primaquine dose, mg/kg	Median reduction in Hb, g/dL	90% prediction interval of reduction in Hb, g/dL*
0	0.25	0	-
	0.4	0	-
0.5	0.25	0.08	0.04-0.12
	0.40	0.12	0.06-0.20
1	0.25	0.14	0.07-0.22
	0.4	0.22	0.12-0.36
1.5	0.25	0.19	0.10-0.31
	0.4	0.30	0.16-0.49
2	0.25	0.23	0.12-0.37
	0.4	0.37	0.20-0.58
3	0.25	0.30	0.17-0.45
	0.4	0.47	0.27-0.70

*Prediction interval resulting from inter-individual variability
