# Supplementary material for: Model-based assessment of the safety of community interventions with primaquine in sub-Saharan Africa
Source: Parasit Vectors. 2021 Oct 9;14:524. doi: 10.1186/s13071-021-05034-4 (PMC8502297; doi:10.1186/s13071-021-05034-4)

Additional file 7: Figure S4. Simulated hemoglobin distributions before and after treatment.

Violin plots of the simulated hemoglobin distributions before treatment and following 0.25 or 0.4 mg/kg primaquine per sub-population used in the World Health Organization's definition of anemia and its severity (**Additional file 1**). The dashed lines represent the cut-offs between the different severity groups.

A. Children 5-11 years of age (25% of the total simulation population of which 6.4% G6PD deficient)

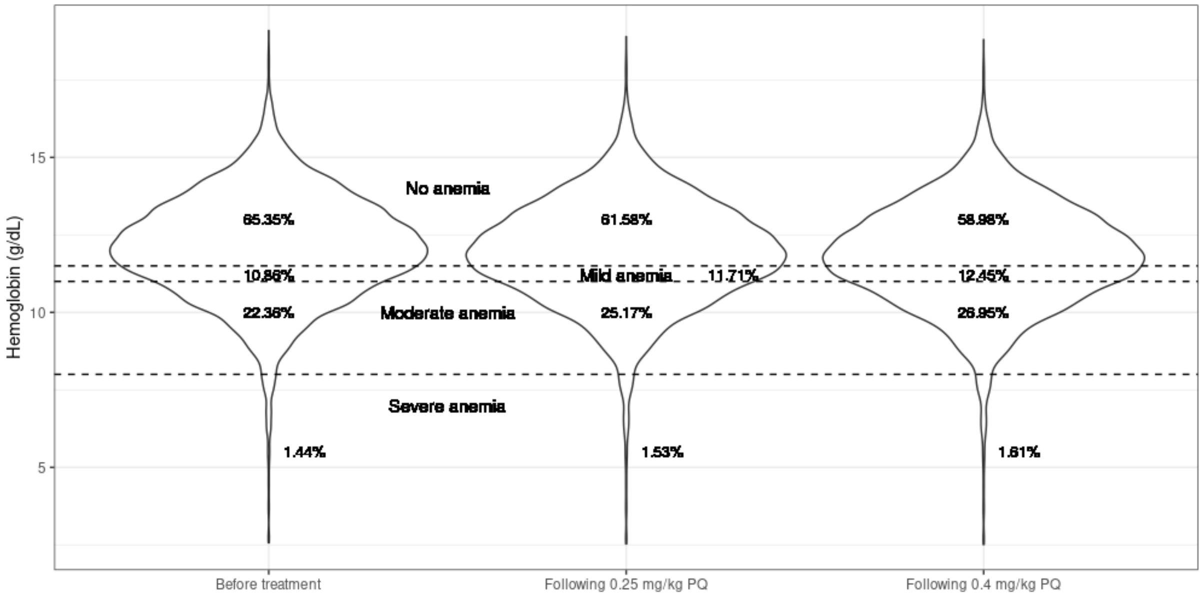

B. Children 12-14 years of age (10% of the total simulation population of which 6.2% G6PD deficient)

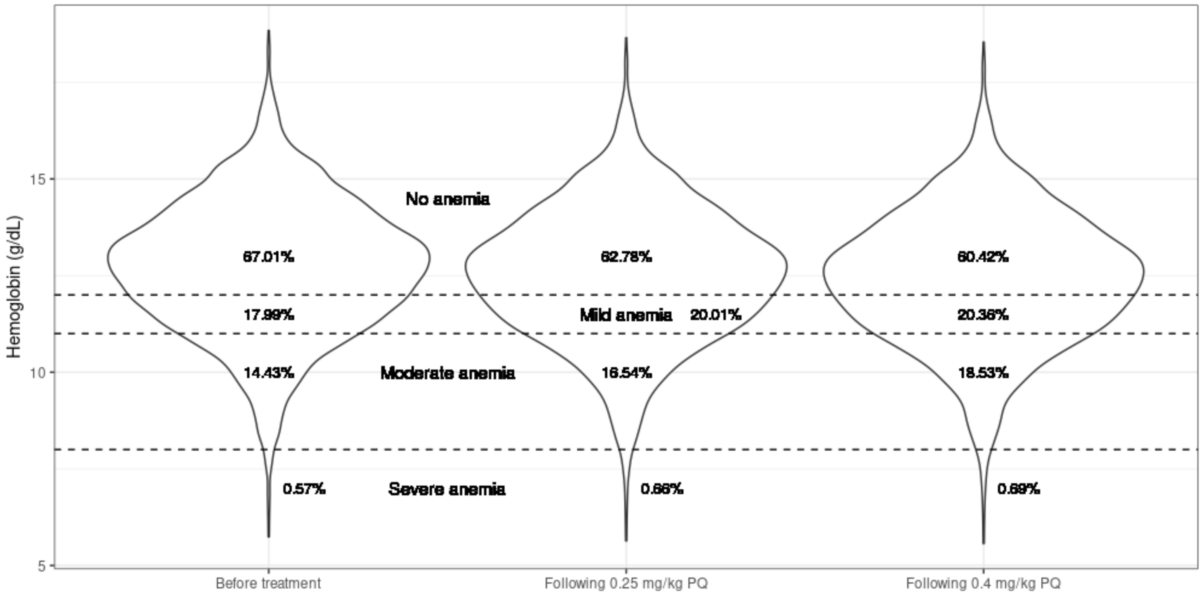

C. Non-pregnant women 15 years of age or older (27% of the total simulation population of which 1.7% G6PD deficient)

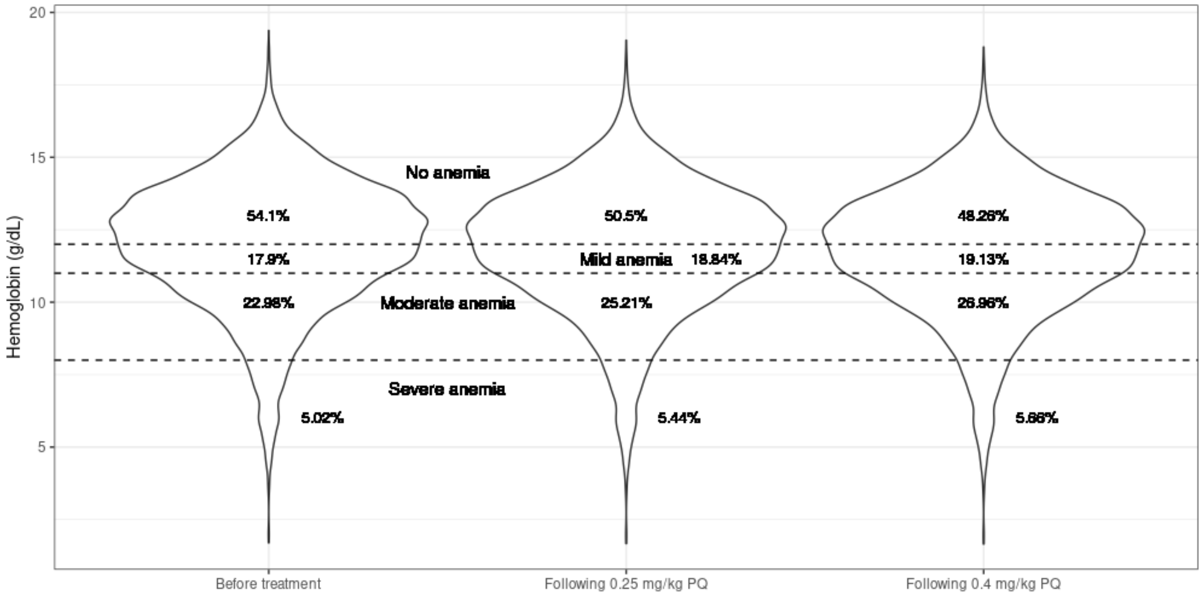

D. Men 15 years of age or older (12% of the total simulation population of which 9.8% G6PD deficient)

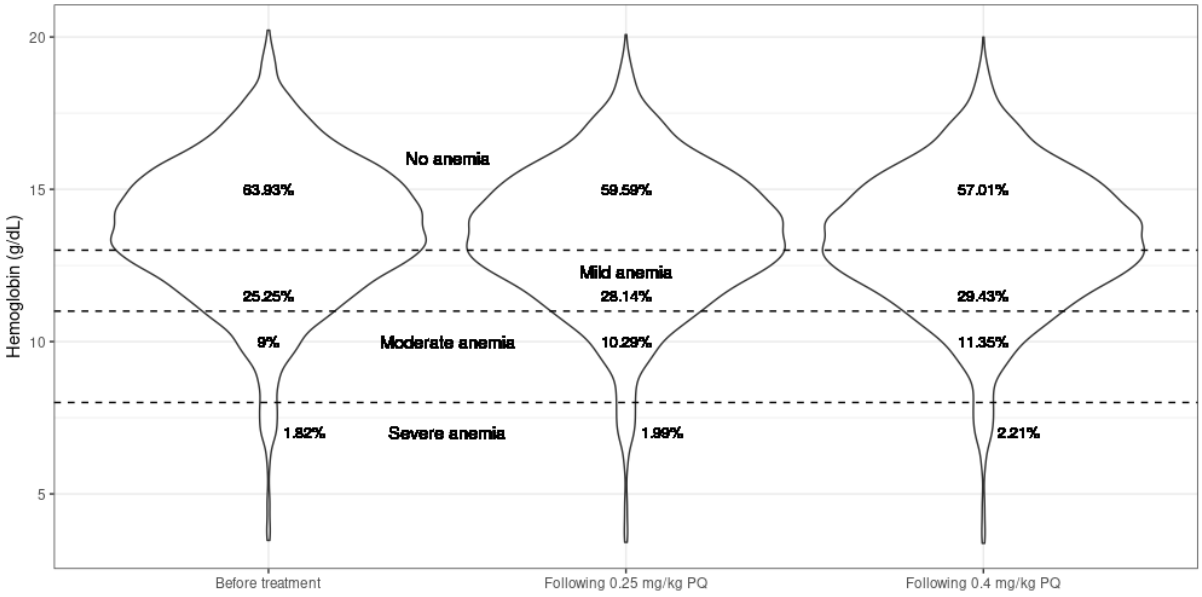

Supplement: Supplementary file 7 — Additional file 7: Figure S4. Simulated hemoglobin distributions before and after treatment. [file 13071_2021_5034_MOESM7_ESM.pdf]
