# Supplementary material for: Model-based assessment of the safety of community interventions with primaquine in sub-Saharan Africa
Source: Parasit Vectors. 2021 Oct 9;14:524. doi: 10.1186/s13071-021-05034-4 (PMC8502297; doi:10.1186/s13071-021-05034-4)

Additional file 8: Figure S5. Distribution of observed pre-treatment hemoglobin levels in the simulation dataset.

Distribution of observed pre-treatment hemoglobin levels for the individuals included in the simulation dataset. The vertical lines correspond to the hemoglobin cut-off values of 7, 7.5 and 8 g/dL. Using a cut-off at 7 g/dL 1.67% of the population is excluded, 2.35% with a cut-off at 7.5 g/dL and 3.35% with a cut-off at 8 g/dL.

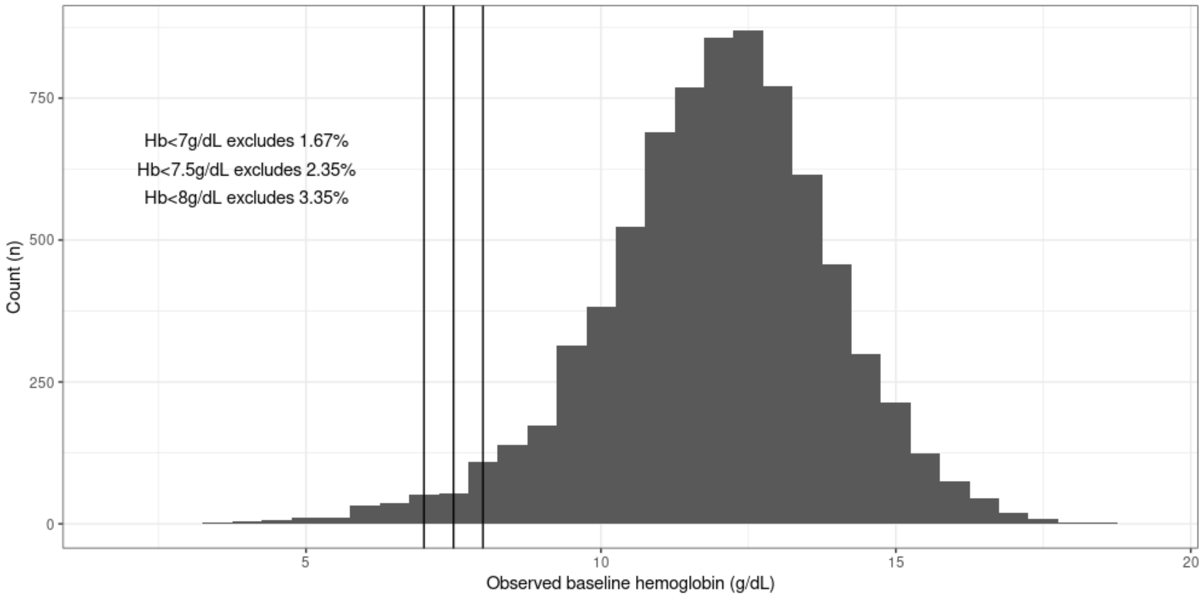

Supplement: Supplementary file 8 — Additional file 8: Figure S5. Distribution of observed pre-treatment hemoglobin levels in the simulation dataset. [file 13071_2021_5034_MOESM8_ESM.pdf]
