# Supplementary material for: Model-based assessment of the safety of community interventions with primaquine in sub-Saharan Africa
Source: Parasit Vectors. 2021 Oct 9;14:524. doi: 10.1186/s13071-021-05034-4 (PMC8502297; doi:10.1186/s13071-021-05034-4)
Supplement: Supplementary file 9 — Additional file 9: Table S3. Predicted prevalence of anemia and its severity after 0.25 and 0.4 mg/kg primaquine when heterozygous females are included in the G6PD-deficient group. [file 13071_2021_5034_MOESM9_ESM.pdf]

Additional file 9: Table S3. Predicted prevalence of anemia and its severity after 0.25 and 0.4 mg/kg primaquine when heterozygous females are included in the G6PD deficient group.

Population	Dosing regimen, mg/kg	No anemia (Hb >11-13 g/dL), %	All anemia grades (Hb <11-13 g/dL), %	Mild anemia (Hb 7-11 to 11-13 g/dL), %	Moderate anemia (Hb 7-8 to 7-11 g/dL), %	Severe anemia (Hb <7-8 g/dL), %
All	0	57.0	43.0	18.3	22.0	2.73
	0.25	52.6	47.4	19.6	24.7	3.02
	0.40	50.0	50.0	20.2	26.5	3.24
G6PD normal	0	58.3	41.7	18.2	20.9	2.60
	0.25	55.0	45.0	19.3	22.8	2.81
	0.40	53.0	47.0	19.9	24.2	2.96
G6PD deficient	0	50.6	49.4	18.9	27.2	3.34
	0.25	41.5	58.5	21.0	33.6	3.43
	0.40	36.0	64.0	21.9	37.6	4.56
